# CNS demyelinating events in primary Sjögren's syndrome: A single-center case series on the clinical phenotype

**DOI:** 10.3389/fneur.2023.1128315

**Published:** 2023-02-16

**Authors:** Ali M. Afzali, Philipp Moog, Sudhakar Reddy Kalluri, Benedikt Hofauer, Andreas Knopf, Jan Stefan Kirschke, Bernhard Hemmer, Achim Berthele

**Affiliations:** ^1^Department of Neurology, School of Medicine, Technical University of Munich, Munich, Germany; ^2^Munich Cluster for Systems Neurology (SyNergy), Munich, Germany; ^3^Department of Nephrology, School of Medicine, Technical University of Munich, Munich, Germany; ^4^Department of Otorhinolaryngology/Head and Neck Surgery, School of Medicine, Technical University of Munich, Munich, Germany; ^5^Department of Otorhinolaryngology/Head and Neck Surgery, Medical University Center, Freiburg, Germany; ^6^Department of Neuroradiology, Technical University of Munich, Munich, Germany

**Keywords:** primary Sjögren's syndrome, sicca, anti-SSA/Ro antibody, anti-SSB/La antibody, salivary gland biopsy, MS, NMOSD

## Abstract

**Objective:**

The study aimed to assess the prevalence, clinical characteristics, and therapeutic outcomes of the central nervous system (CNS) demyelinating disease in a large cohort of primary Sjögren's syndrome (pSS).

**Methods:**

This is an explorative cross-sectional study of patients with pSS seen in the departments of rheumatology, otorhinolaryngology, or neurology of a tertiary university center between January 2015 and September 2021.

**Results:**

In a cohort of 194 pSS patients, 22 patients had a CNS manifestation. In this CNS group, 19 patients had a lesion pattern suggestive of demyelination. While there were no obvious differences in the patients' epidemiological disposition or rate of other extraglandular manifestations, the CNS group differed from the remaining patients with pSS by having less glandular manifestations but a higher seroprevalence for anti-SSA/Ro antibodies. Notably, patients with CNS manifestations were often diagnosed with multiple sclerosis (MS) and treated as such, although age and disease course were atypical of MS. Many first-line MS agents were ineffective in these “MS look-alikes”; however, the disease course was benign with B-cell-depleting agents.

**Conclusion:**

Neurological symptoms of pSS are common and clinically manifest mainly as myelitis or optic neuritis. Notably, in the CNS, the pSS phenotype can overlap with MS. The prevailing disease is crucial since it has a major impact on the long-term clinical outcome and the choice of disease-modifying agents. Although our observations neither confirm pSS as a more appropriate diagnosis nor rule out simple comorbidity, physicians should consider pSS in the extended diagnostic workup of CNS autoimmune diseases.

## Introduction

Primary Sjögren's syndrome (pSS) is an autoimmune disease of the exocrine glands characterized by sicca syndrome and a corresponding chronic lymphocytic infiltration. In the course of the initial diagnostic workup, staging of further organ manifestations is of great importance since it has prognostic relevance and thus influences the choice of disease-modifying agents. In pSS, extraglandular manifestation is a common phenomenon and a risk factor for increased mortality (1–4).

Although extraglandular symptoms of pSS are well described (5–9), pSS is seldom considered an important differential diagnosis of autoimmune demyelinating disorders. This may be particularly relevant for multiple sclerosis (MS), where the McDonald diagnostic criteria allow a definite diagnosis only if there is no better explanation (10). Due to overlapping autoantibody patterns, pSS may also obscure the proper diagnosis of neuromyelitis optica spectrum disease (NMOSD) (11).

Establishing an appropriate diagnosis early on is also relevant when it comes to immunotherapy and the prediction of long-term clinical outcomes (12, 13). Unfortunately, there are no prospective intervention studies in pSS with extraglandular symptoms from which evidence-based treatment recommendations can be derived. Only retrospective analyses indicate a benefit of B-cell depletion (14, 15). Among other extraglandular symptoms, broader vigilance and appreciation of neurological manifestations of pSS may help identify patients at earlier stages of their disease and guide putative therapeutic regimens.

Thus, we aimed at investigating the prevalence and characteristics of neurological manifestations of pSS in a single-center cross-sectional approach. In the present study, we retrospectively analyzed all pSS cases seen in our university hospital between January 2015 and September 2021.

## Materials and methods

### Patient cohort

We conducted this observational cross-sectional monocentric study at the University Hospital “Klinikum rechts der Isar” of the Technical University, Munich, Germany, a tertiary center for neurological, rheumatologic, and otorhinolaryngology (ENT) diseases. We identified all patients with pSS seen between January 2015 and September 2021 in the departments of rheumatology, ENT, or neurology. Electronic files were screened for the corresponding code of Sjögren's syndrome (M35.0-) according to the 10th revision of the International Classification of Diseases (ICD-10). Part of the data obtained from this cohort was also integrated into the *Big Data Sjögren Consortium* (16).

Primary Sjögren's syndrome was diagnosed according to the classification criteria proposed by the *International Sjögren's syndrome Criteria Working Group* and approved by the *American College of Rheumatology (ACR)* and the *European League Against Rheumatism (EULAR)* (7). We excluded patients with a competing classifiable rheumatic disease in terms of an overlap syndrome.

We assessed systemic disease activity with the *EULAR Sjögren's syndrome disease activity index* (ESSDAI) (5, 17). This index comprises 12 domains and is regarded as the gold standard for scientific studies on pSS. However, due to the retrospective nature of our study, data sets on ESSDAI domains in our patients were often incomplete. Therefore, we narrowed *the clinical domains* to dichotomous variables (absent vs. present). CNS manifestations were classified based on further clinical and paraclinical data [evoked potentials/nerve conduction studies, magnetic resonance imaging (MRI), and CSF (cerebrospinal fluid) analysis].

### Serum analyses

We analyzed anti-SSA/Ro and anti-SSB/La antibodies with an enzyme-linked immunosorbent assay (ELISA, Orgentec Diagnostika, Mainz, Germany). Anti-aquaporin-4 IgG antibodies (AQP4-Ab) were determined by a commercially available cell-based assay (Euroimmun Perkin Elmer, Luebeck, Germany) and anti-myelin oligodendrocyte glycoprotein (MOG) antibody (MOG-Ab) with an in-house cell-based assay, as described previously (18).

### MRI analyses

The T2-weighted whole brain and spinal MRI scans of individual patients were evaluated in a blinded manner. The distribution of T2 hyperintensities was analyzed manually in a pattern-based approach. The spinal cord was analyzed in total and divided into cervical and thoracolumbar regions according to the anatomical nomenclature.

### Statistical analysis

We used Fisher's test for nominal variables, Student's *t*-test for age/time comparisons, and Mann–Whitney *U*-test for ESSDAI parameters. We did not adjust for multiple testing due to the exploratory retrospective study design. We performed all statistical tests by using IBM SPSS Statistics for Windows (version 23.0, Armonk, NY, IBM Corp).

## Results

**Figure 1:**
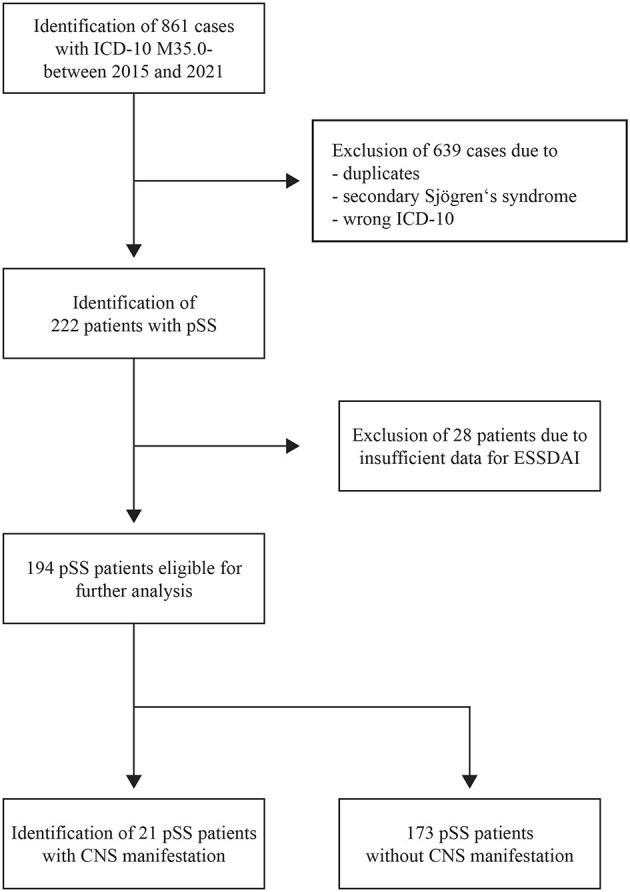
PRISMA flow diagram. PRISMA flow diagram showing the different steps of the systematic review of patients' electronic files. CNS, central nervous system; ESSDAI, EULAR Sjögren's syndrome disease activity index; ICD-10, 10th revision of the International Classification of Disease; pSS, primary Sjögren's syndrome.

We identified 861 cases with a diagnosis of M35.0- (ICD-10), of which 639 cases were excluded because they were duplicates, had secondary Sjögren's syndrome, or their ICD codes were incorrect. Altogether, 222 patients with pSS were treated in our facilities between January 2015 and September 2021. Due to insufficient data for our modified ESSDAI, only 194 patients were available for further analysis (see Figure 1). Epidemiological data and serological and clinical parameters are presented in Table 1.

**Table 1:** Demographic data and baseline clinical characteristics of pSS patients with and without CNS manifestation.

	**Control group**	**CNS group**	***p*-Value (CNS vs. total)**
Sex female, *n* (%)	152/173 (87.9)	14/19 (73.7)	0.147
Age at diagnosis, years; median [IQR]	55.5 [43–68]	51 [46.5–60]	0.374
Age at symptom onset, years; median [IQR]	54 [37.75–65]	45 [31–56]	0.084
Time to pSS diagnosis, years; median [IQR]	1 [0–3]	3 [1.3–15.5]	**0.009**
Positive ESSDAI items; median [IQR]	3 [2–4]	3 [3–4]	0.201
Constitutional symptoms; *n* (%)	26/173 (15.0)	5/19 (26.3)	0.200
Lymphadenopathy; *n* (%)	26/173 (15.0)	1/19 (5.3)	0.483
Salivary gland swelling; *n* (%)	52/173 (30.0)	2/19 (10.5)	0.105
Xerostomia; *n* (%)	155/173 (89.6)	8/19 (42.1)	**< 0.00001**
Keratoconjunctivitis sicca; *n* (%)	147/173 (84.9)	8/19 (42.1)	**0.0001**
Arthralgia/arthritis; *n* (%)	45/173 (26.0)	6/19 (31.6)	0.592
Skin; *n* (%)	22/173 (12.7)	2/19 (10.5)	>0.999
Lung; *n* (%)	8/173 (4.6)	0/19 (0)	>0.999
Kidney; *n* (%)	11/173 (6.4)	2/19 (10.5)	0.622
Myositis; *n* (%)	9/173 (5.2)	0/19 (0)	0.603
PNS; *n* (%)	12/173 (6.9)	3/19 (15.8)	0.173
Hematological; *n* (%)^a^	24/173 (13.9)	8/19 (42.1)	**0.01**
C3 low; *n* (%)^b^	18/117 (15.4)	5/15 (33.3)	0.147
C4 low; *n* (%)^b^	11/117 (9.4)	3/15 (20.0)	0.199
IgG altered, *n* (%)	6/36 (16.7)	4/18 (22.2)	0.715
ANA IF positive; *n* (%)^c^	156/170 (91.7)	18/19 (94.7)	>0.999
Anti-SSA/Ro positive; *n* (%)^c^	85/173 (49.1)	15/19 (78.9)	**0.016**
Anti-SSB/La positive; *n* (%)^c^	48/169 (28.4)	7/19 (36.8)	0.436
Salivary gland biopsy performed; *n*	117/173 (67.6)	16/19 (80.0)	0.191
Biopsy positive; *n* (%)^d^	112/117 (95.7)	14/16 (87.5)	0.199
Seronegative & biopsy positive; *n* (%)	73/159 (45.9)	3/19 (15.8)	**0.014**

Differences were assessed with Fisher's test for nominal variables, Student's t-test for age/time comparisons, and Mann–Whitney U-tests for ESSDAI parameters.

^a^Anemia and/or abnormal leukocyte counts.

^b^C3 < 90 mg/dl, C4 < 10 mg/dl.

^c^ANA immunofluorescence (IF) positive >1:120, anti-SSA/Ro >25 U/ml, and anti-SSB/La >25 U/ml.

^d^Salivary gland biopsy positive with a Chisholm–Mason focus score of ≥1.

CNS, central nervous system; IgG, Immunoglobulin G; IQR, interquartile range; na, not available; PNS, peripheral nervous system; pSS, primary Sjögren's syndrome. *P* values below the significance level of < 0.05 are marked in bold.

### Patient's characteristics—pSS patients with neurological manifestations have less frequent signs of glandular disease and are more often seropositive for anti-SSA/Ro antibodies

The involvement of the CNS was defined by the presence of focal neurological deficits indicative of CNS disorder and/or supported by morphological alterations of the brain and/or the spinal cord on MRI. Twenty-one patients had CNS involvement (21/194, 10.8%) and of these, 19 patients presented with a CNS lesion pattern compatible with demyelination based on radiological findings and/or CSF parameters (referred to as the “CNS group”). A total of 173 pSS patients without CNS manifestations were available for comparison (the “control group”; Table 1).

In the CNS group, 14 patients were female (14/19, 73.7%). The median age [interquartile range (IQR)] at symptom onset and diagnosis was 45 years (31–56) and 51 years (46.5–60), respectively. The median time of diagnosis was 2 years longer in the CNS group than in the control group (*p* = 0.009). In the CNS group, neurological deficits were the first manifestation of pSS in 16 patients (16/19; 84.2%), and in 14 patients (14/19, 73.7%), they were the primary reasons to consult a physician.

Only 2 of 19 CNS patients (2/19, 10.5%) reported sicca symptoms [xerostomia and/or keratoconjunctivitis sicca (KCS)] at first encounter, with one of them already having a history of known pSS. Eight patients (8/17, 47.0%) developed sicca symptoms in the course of the disease. Sicca symptoms occurred more frequently in the control group [control vs. CNS group; xerostomia 155/173 (89.6%) vs. 8/19 (42.1%) with *p* < 0.00001; KCS 147/173 (84.9%) vs. 8/19 (42.1%) with *p* = 0.0001)]. Regarding other signs of systemic pSS, only hematological alterations mainly comprising anemia were more present in the CNS group (*p* = 0.01; Table 1).

In the CNS group, seroprevalence for anti-SSA/Ro was higher leading to a definitive diagnosis of pSS [85/173 (49.1%) vs. 15/19 (78.9%), *p* = 0.016], while the control group had more seronegative patients requiring a salivary gland biopsy (SGB) for their pSS diagnosis [73/159 (45.9%) vs. 3/19 (15.8%), *p* = 0.014].

### Central nervous system manifestations—Myelitis and optic neuritis predominate

Central nervous system (CNS) symptoms consisted mainly of sensorimotor syndromes due to myelitis or visual impairment due to optic neuritis—both accompanied or not by active/non-active brain lesions in the MRI.

**Figure 2:**
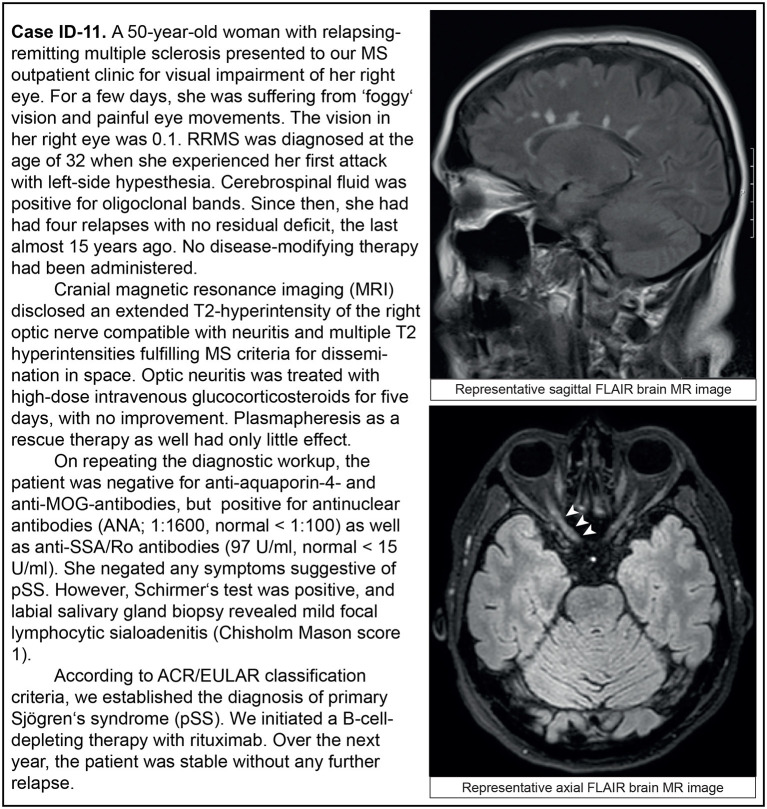
Representative clinical case vignette of patient ID-11.

**Figure 3:**
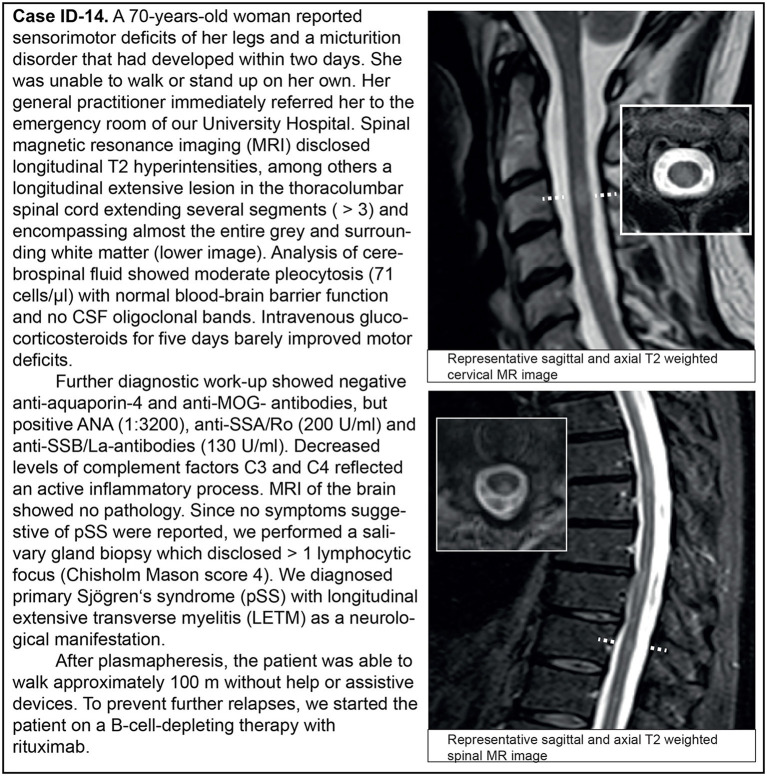
Representative clinical case vignette of patient ID-14.

We analyzed the clinical phenotypes, serological profiles, and lesion patterns on whole brain and spinal MRI. Myelitis was the index event in 13 patients (13/19; 68.4%; Table 2A, Figure 2), optic neuritis occurred in five patients (5/19; 26.3%; Table 2A, Figure 3), and four patients had both in the further course of the disease (4/19; 21.1%; Table 2A). MRI scans at the index event revealed corresponding T2 hyperintensities in all patients with myelitis (13/13, 100%) and three patients with optic neuritis (3/5, 60%). Of patients with a history of optic neuritis, MRI revealed T2 hyperintensities in six patients (6/8, 75%), three extended throughout the optic nerve (3/8, 37.5%), two were located in the anterior part of the optic nerve (2/8, 25%), and two affecting both optic nerves simultaneously (2/8, 25%). Notably, 13 patients had signs of T2 hyperintensities on index brain MRI consistent with demyelination (13/19, 68.4%). In total, 14 out of 19 patients with CNS manifestation reported sensory deficits (14/19, 73.7%), five patients each had visual disturbances or bladder dysfunction (5/19, 26.3% each), and seven patients had motor deficits (7/19, 36.8%). Most patients accrued some degree of neurological disability. Eleven patients were presented to us initially during a clinical attack. Of these, eight patients were treated with steroids with four being responsive (4/8, 50%). Only one patient had recovered entirely from her symptoms (1/19, 5.3%); in all others, residual deficits remained. In three patients, the disease course was progressive (3/19, 15.8%).

**Table 2:** Clinical characteristics of pSS patients with CNS manifestations.

	**Gender** **(F/M)**	**Age at diagnosis (year)**	**Age at symptom onset (year)**	**Sicca**	**Time to sicca (y)**	**Known pSS diagnosis**	**Myelitis/** **ON**	**Attacks/** **year**	**Last** **EDSS**	**No. of** **ESSDAI items**
**A**										
ID-1	F	46	30	+	14	–	Myelitis	0.5	2.0	3
ID-2	F	49	48	–	–	–	ON	0.2	2.0	2
ID-3	F	43	39	+	6	–	Both	0.3	2.5	4
ID-4	F	31	29	–	–	+	Both	1 attack	1.0	6
ID-5	M	58	57	–	–	–	Myelitis	1 attack	7.0	5
ID-6	F	62	45	+	18	–	Myelitis	Progressive	3.5	2
ID-7	F	57	55	+	3	–	Myelitis	2.5	3.5	4
ID-8	F	51	30	+	0	–	Myelitis	0.05	3.0	3
ID-9	F	55	53	–	–	–	Myelitis	0.25	2.0	1
ID-10	F	51	20	+	31	–	Both	0.33	2.0	8
ID-11	F	50	32	–	–	–	ON	3.0	3.0	3
ID-12	F	64	59	+	5	–	ON	0.25	0	5
ID-13	M	63	63	–	–	–	Myelitis	Progressive	5.0	3
ID-14	F	70	69	–	–	–	Myelitis	1.0	7.0	3
ID-15	M	28	28	–	na	–	Myelitis	0.25	2.0	1
ID-16	F	54	37	+	18	–	Both	1.0	4.5	3
ID-17	M	46	45	–	–	–	ON	1 attack	2.0	1
ID-18	M	66	62	+	0	–	Myelitis	progressive	2.0	5
ID-19	F	47	45	+	2	–	Myelitis	0.75	2.0	4
%	73.7 (14/19)			52.6 (10/19)		5.3 (1/19)				
Median [IQR]		51 [46.5–60]	45 [31–56]		5.5 [2.25–17]			0.33 [0.25–1]	2.0 [2.0–3.5]	3 [3–4]
**B**											
ID-1	+/+	na	na	+	na	na	na	+	+	+	–
ID-2	+/–	+	4°	+	–	–	+	+	–	–	–
ID-3	–/–	+	4°	+	–	–	na	+	+	+	–
ID-4	+/+	+	4°	+	–	–	+	–	+	–	–
ID-5	+/–	+	4°	+	–	–	na	+	–	–	–
ID-6	+/–	–	3–4°	+	na	–	na	+	+	+	–
ID-7	+/–	na	3°	+	–	–	na	+	+	+	–
ID-8	+/+	+	na	–	–	–	na	+	+	+	–
ID-9	+/–	–	4°	+	na	–	na	+	+	+	–
ID-10	–/–	+	3°	–	–	na	na	+	+	+	–
ID-11	+/–	+	1°	+	–	–	+	+	+	+	–
ID-12	–/–	na	3°	–	na	–	–	–	+	+	–
ID-13	+/–	+	0	–	–	na	na	+	–	–	–
ID-14	+/+	na	4°	–	–	–	na	+	+	+	–
ID-15	+/–	na	na	+	na	na	na	+	+	+	–
ID-16	+/+	+	4°	+	+	–	na	+	+	–	+
ID-17	–/+	–	3°	na	–	–	–	–	+	–	–
ID-18	+/–	na	0	+	na	–	na	+	–	–	–
ID-19	+/+	na	4°	+	+	na	na	+	+	–	+
%	84.2 (16/19)	75 (9/12)	3.75 [3–4]	72.2 (13/18)	15.4 (2/13)	0 (0/14)	60 (3/5)	84.2 (16/19)	78.9 (15/19)	57.9 (11/19)	10.5 (2/19)

Demographic, clinical (both A), and diagnostic parameters (B) of the CNS group. Data are shown as either proportion with absolute numbers or median with interquartile range (IQR).

^a^≥ two oligoclonal bands (OCB) in the cerebrospinal fluid (CSF).

^b^AQP4-Ab positive >1:10.

CNS, central nervous system; CSF, cerebrospinal fluid; EDSS, Expanded disability status scale; IQR, interquartile range; MS, multiple sclerosis; na, not available; NMOSD, neuromyelitis optica spectrum disease; OCB, oligoclonal bands; ON, optic neuritis; pSS, primary Sjögren's syndrome; T2+, T2 hyperintensities in MRI scans.

We observed isolated myelitis or optic neuritis without further morphological correlation of demyelination on whole brain and whole spine MRI in five patients (5/19, 26.3%). Of these, one patient had already been diagnosed with pSS, and in the remaining four patients, initial diagnostic workup led to the detection of anti-SSA/Ro and/or anti-SSB/La and consequently to the diagnosis of pSS. Thus, isolated myelitis or optic neuritis was classified as a demyelinating event associated with pSS. This association turned out to be less straightforward in all other patients whose MRI scans revealed additional asymptomatic lesions that were disseminated in space and thus suggestive of chronic inflammatory CNS diseases such as MS or NMOSD.

### Primary Sjögren's syndrome (pSS) patients with MS or NMOSD

**Figure 4:**
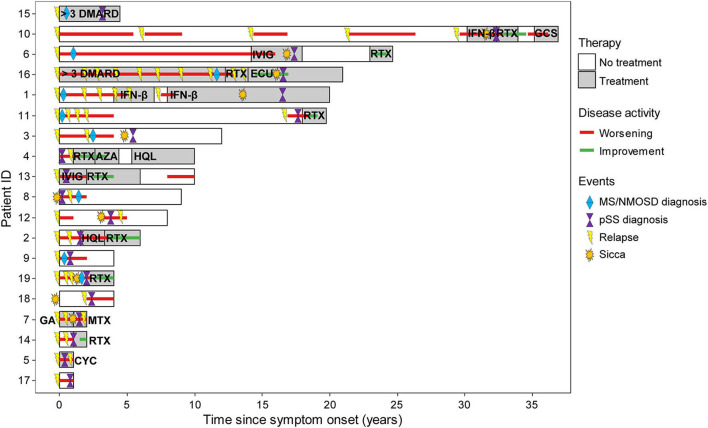
Individual disease course in the CNS group. The Swimmer plot showing individual disease course and treatment response to immunotherapy since disease onset. The color of any text within bars indicates treatment. The colored lines indicate disease activity. Specific events were inserted into the bars as indicated. In the medical records of two patients, treatment history was summarized as >3 disease-modifying antirheumatic drugs (DMARD) and not further specified. AZA, azathioprine; CNS, central nervous system; CYC, cyclophosphamide; ECU, eculizumab; GA, glatiramer acetate; GSC, glucocorticosteroid; HQL, hydroxychloroquine; IFN-β, interferon β; IVIG, intravenous immunoglobulin; MS, multiple sclerosis; MTX, methotrexate; NMOSD, neuromyelitis optica spectrum disease; RTX, rituximab.

A total of eight patients had been diagnosed with MS and were treated as such, and three more patients fulfilled the McDonald's criteria of 2017 (10). Notably, only 8 of these 11 patients (8/11, 72.7%) were positive for CSF-specific oligoclonal bands (OCB) with 13 of the total CNS group being OCB positive (13/18; 72.2%). Two patients were positive for anti-AQP4 antibodies (2/13, 15.4%) and diagnosed with NMOSD according to the IPND criteria of 2015 (11). Of 14 tested patients, none were positive for anti-MOG antibodies (0/14). Another three patients fulfilled the diagnostic criteria for AQP4 antibody-negative NMOSD. Both patients with anti-AQP4-antibody-positive NMO presented with an initial demyelinating event that left debilitating residual deficits. After initiating disease-modifying therapy based on CD20 depletion, further relapses could be prevented (Figure 4). The three other patients had a history of both myelitis and optic neuritis, which was rather mild compared with prognostic outcomes from observational NMOSD studies.

In all these cases, the diagnosis of pSS was finally made in the course of the disease either when the disease remained active under therapy (e.g., recurring optic neuritis) or when the diagnosis was facilitated by signs of systemic disease or elevated ANA titers. In 10 patients (10/19; 52.6%), symptoms suggestive of sicca syndrome did not occur until a median of 5.5 years (2.25–17) after the onset of neurological symptoms. Two patients reported Raynaud's phenomenon (2/19, 10.5%), and six patients had arthralgia (6/19, 31.6%). Five patients from the CNS group without subjective signs of sicca syndrome had a positive Schirmer's test (5/9, 55.6%), and in four of these five patients, salivary gland biopsy (SGB) disclosed pathognomonic lymphocytic infiltrates with a median Chisholm–Mason focus score of 4 (3, 4). Similar histological results after SGB could be obtained in the remaining CNS group (14/16, 87.5%).

### Immunotherapy

A total of eight patients were started on disease-modifying antirheumatic drugs (DMARD) after pSS diagnosis, and ongoing immunomodulatory treatment was adjusted in three patients with prior MS diagnosis. Of note, five patients receiving first-line agents approved for MS had ongoing disease activity (Figure 4). Most successful was B-cell depletion with rituximab with a positive response (stable disease or improvement) in eight out of nine patients (8/9, 88.9%). Three patients (3/19, 15.8%) refused long-term immunosuppression, and two patients (2/19, 10.5%) did not require immunomodulatory therapy due to stable disease. Four patients required more than three drugs (4/19, 21%), and six patients deteriorated at least once in the course of the disease (6/19, 31.6%), requiring apheresis therapy as an acute rescue intervention. The median EDSS (*extended disability status scale*) was 2.0 (IQR 2.0–3.5) with a disease duration of 9 years (6–21.5) and a yearly attack rate of 0.33 (0.25–1.0; Table 2) indicating an overall disabling disease course with permanent deficits in our pSS patients with CNS manifestations.

### Lesion pattern along with the spinal cord suggests other differential diagnoses than MS

While spinal cord lesions in MS are supposed to occur in the periphery of the spinal cord and usually extend over less than three vertebral segments, lesions in NMOSD and other systemic autoimmune may preferentially affect the center of the spinal cord and may exceed more than three vertebral segments (19). We took a pattern-based approach identifying the localization of T2-hyperintense lesions within the spinal cord of all patients from the CNS group and identified a similar pattern distribution as proposed by Goh et al. (19).

**Figure 5:**
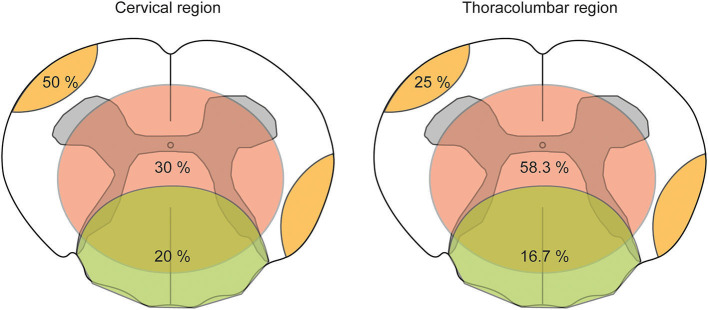
Pattern-based visualization of axial T2 hyperintensities distributed along with the spinal cord in the CNS group. The pattern-based approach of visualizing the distribution of axial T2 hyperintensities analyzed on axial T2-weighted magnetic resonance imaging (MRI) along with the spinal cord. The spinal cord was divided into a cervical and thoracolumbar regions as to neuroanatomical nomenclature. Within a schematic drawing of an axial slice of the spinal cord, three types of lesion patterns are shown: peripheral lesion (orange color), central lesion (red color), and dorsal lesion (green color). Numbers within colored drawings indicate the frequency among all lesions counted.

A total of three patients were devoid of T2 hyperintensities along with the spinal cord (3/19, 15.8%). Of the remaining patients with spinal cord lesions, eight had at least two or even more T2 hyperintensities (8/16, 50%), and we could identify five patients with T2 hyperintensities that exceeded more than three vertebral segments (5/16, 31.25%) fulfilling the criteria for longitudinal extensive transverse myelitis (LETM) (20). On sagittal images, lesions were distributed over the entire spinal cord without a distinct site of predilection. On axial images, T2 hyperintensities were distributed mainly in three patterns. We observed that lesions were localized at the periphery affecting the ascending and descending tracts, central lesions covering the gray matter and its surroundings, and lesions at the dorsal region involving the lemniscal tracts. Most lesions were localized in the central region (12 out of 27 lesions in total, 44.4%), followed by lesions in the periphery (10/27, 37%) and the dorsal region (5/27, 18.5%). The relative distribution of lesions in the cervical and thoracolumbar regions of the spinal cord is presented in Figure 5.

## Discussion

Rheumatological diseases are challenging in the differential diagnosis and therapy of autoimmune diseases of the central nervous system. It becomes even more puzzling when the diagnostic criteria of chronic inflammatory CNS diseases such as MS or NMOSD are fulfilled, but systemic autoimmune diseases such as pSS coincide. For this reason, we set out to retrospectively summarize all pSS cases with neurological deficits that could be attributed to CNS lesions and compare them with pSS patients without neurological deficits.

Extraglandular manifestations of pSS are a well-known phenomenon (5, 7), and neurological symptoms are quite common in patients with pSS (21–27). In our study, almost every eighth patient was affected. In patients with an established diagnosis of pSS, staging for neurological symptoms is a part of the routine examination, and neurological symptoms are rapidly recognized as a systemic manifestation of the disease. However, the final diagnosis of pSS in patients with an initial demyelinating event takes quite a long time. In our cohort, the final diagnosis took almost 2 years longer.

Some of these patients were provisionally diagnosed with MS and eventually diagnostic uncertainty led to further investigation and thus the diagnosis of pSS. Older age than usual for MS might have raised concerns, and either persistent symptoms or deterioration under therapy have led to more targeted investigations. Finally, signs of systemic disease, elevated ANA levels, and/or a positive Schirmer's test have prompted SGB in our patients. Admittedly, high ANA levels are rather unspecific and also common in MS (28, 29). Therefore, the serological, clinical, and paraclinical overlap hampers a straightforward and reliable differentiation of these two disease entities. While pSS has received considerable attention in the differential diagnosis of isolated optic neuritis and myelitis (20, 30, 31), it is less consistently considered in chronic inflammatory disorders that primarily involve the CNS.

Still, the question remains unresolved whether MS or its phenotype and pSS *or its serology* simply coincided in our CNS cohort. In this respect, it is interesting to note that despite sicca symptoms being rare at the beginning, almost all of our CNS patients had serological and histological evidence of an inflammatory affection of salivary glands typical of pSS. Therefore, at least an accompanying pSS is to be assumed, and data from genome-wide association studies suggest at least a genetic overlap (32). Given that the diagnosis of MS can only be made when there is no better explanation (10), there were also hints calling even the diagnosis of MS into question. CSF findings were not decisive with only 13 out of 18 patients (13/18, 72.7%) being positive for OCB. While the MRI pattern of cerebral lesions was compatible with MS, spinal lesion patterns were rather atypical for MS, and finally, standard MS modifying drugs were partially ineffective in our group of patients.

Although out of reach, an unequivocal attribution of the inflammatory CNS lesions' etiology would impact choosing the appropriate therapy. Insufficient disease control can have a reciprocal influence on coinciding autoimmune diseases (33–35), and published data suggest that extraglandular manifestation of pSS is associated with increased mortality (4, 14). For extraglandular manifestations of pSS, treatment guidelines recommend cyclophosphamide or rituximab in combination with corticosteroids (1, 6, 12, 13). In line with previously published data (14, 15), B-cell depletion proved effective in our patients when MS agents such as ocrelizumab or ofatumumab were not yet available.

However, selection bias is a relevant matter in our cohort, which was rather different compared with typical MS patients with respect to age and onset. This precludes the suggestion that SGB should be a part of the standard MS workup. Instead, our data indicate that in patients with a phenotype compatible with both disease or refractory to MS disease-modifying drugs, a SGB is advisable — as long as there is no other separating biomarker, especially in patients who develop sicca symptoms or signs of systemic disease. Accordingly, SGB is recommended broadly in clinical practice (7, 36). It does not only help to confirm a diagnosis, especially in seronegative patients but also has prognostic relevance (36, 37).

There are further restrictions on our data that we have to mention. As a tertiary center, selection bias needs to be considered so that we attract more severely affected patients. Due to the limited cohort size and the exploratory study design, the statistical analysis has not been adjusted for multiple testing. Diagnostic tests to detect sicca symptoms were not performed routinely, as every patient with pSS did not receive an MRI scan or an electrophysiological workup. Morreale et al. proposed an association of pSS with headache syndromes and neurocognitive/psychological disorders (38), and a recent study has focused on cognitive dysfunction and dementia in pSS (39, 40), which we did not address either. Moreover, we were not able to recapitulate each individual treatment decision hindering a more systematic analysis.

Taken together, we observed a considerably high rate of neurological manifestations in our highly selected cohort of consecutive patients with pSS. Cases are segregated into distinct CNS phenotypes, which can be relevant to the extended differential diagnosis of neurological autoimmune diseases. Second, a thorough evaluation of applied diagnostic criteria such as the McDonald's criteria may be necessary for the individual to avoid misdiagnosis. Thus, further studies and insights into the pathophysiology are required to overcome the stated obstacles and pave the way for refined diagnostic procedures and new therapeutic approaches.

## Data availability statement

The raw data supporting the conclusions of this article will be made available by the authors, without undue reservation.

## Ethics statement

The Ethics Committee of the Technical University Munich has approved the study (file number 132/19 S-SR). The patients/participants provided their written informed consent to participate in this study. Written informed consent was obtained from the individual(s) for the publication of any potentially identifiable images or data included in this article.

## Author contributions

AA: conceptualization, methodology, formal analysis, investigation, data curation, writing—original draft, and visualization. PM: data curation, formal analysis, and writing—review and editing. SK, BHo, and JK: data curation and writing—review and editing. AK and BHe: writing—review and editing. AB: conceptualization, methodology, writing—review and editing, supervision, and project administration. All authors contributed to the article and approved the submitted version.

## References

[1] Carsons SE Vivino FB Parke A Carteron N Sankar V Brasington R. Treatment guidelines for rheumatologic manifestations of sjogren's syndrome: use of biologic agents, management of fatigue, and inflammatory musculoskeletal pain. Arthritis Care Res. (2017) 69:517–27. 10.1002/acr.22968 27390247

[2] Flores-Chavez A Kostov B Solans R Fraile G Maure B Feijoo-Masso C. Severe, life-threatening phenotype of primary Sjogren's syndrome: clinical characterisation and outcomes in 1580 patients (GEAS-SS Registry). Clin Exp Rheumatol. (2018) 36:121–9. 30156546

[3] Huang H Xie W Geng Y Fan Y Zhang Z. Mortality in patients with primary Sjogren's syndrome: a systematic review and meta-analysis. Rheumatology. (2021) 60:4029–38. 10.1093/rheumatology/keab364 33878179

[4] Singh AG Singh S Matteson EL. Rate, risk factors and causes of mortality in patients with Sjogren's syndrome: a systematic review and meta-analysis of cohort studies. Rheumatology. (2016) 55:450–60. 10.1093/rheumatology/kev354 26412810 PMC5009445

[5] Seror R Ravaud P Bowman SJ Baron G Tzioufas A Theander E. EULAR Sjogren's syndrome disease activity index: development of a consensus systemic disease activity index for primary Sjogren's syndrome. Ann Rheum Dis. (2010) 69:1103–9. 10.1136/ard.2009.110619 19561361 PMC2937022

[6] Price EJ Rauz S Tappuni AR Sutcliffe N Hackett KL Barone F. The British Society for Rheumatology guideline for the management of adults with primary Sjogren's syndrome. Rheumatology. (2017) 56:e24–48. 10.1093/rheumatology/kex163 28957550

[7] Shiboski CH Shiboski SC Seror R Criswell LA Labetoulle M Lietman TM. 2016 American College of Rheumatology/European League Against Rheumatism classification criteria for primary Sjogren's syndrome: a consensus and data-driven methodology involving three international patient cohorts. Ann Rheum Dis. (2017) 76:9–16. 10.1136/annrheumdis-2016-210571 27789466

[8] Brito-Zerón P Acar-Denizli N Ng W-F Zeher M Rasmussen A Mandl T. How immunological profile drives clinical phenotype of primary Sjögren's syndrome at diagnosis: analysis of 10,500 patients (Sjögren Big Data Project). Clin Exp Rheumatol. (2018) 36:102–12. 30156539

[9] Retamozo S Acar-Denizli N Rasmussen A Horvath IF Baldini C Priori R. Systemic manifestations of primary Sjogren's syndrome out of the ESSDAI classification: prevalence and clinical relevance in a large international, multi-ethnic cohort of patients. Clin Exp Rheumatol. (2019) 37:97–106. 31464664

[10] Thompson AJ Banwell BL Barkhof F Carroll WM Coetzee T Comi G. Diagnosis of multiple sclerosis: 2017 revisions of the McDonald criteria. Lancet Neurol. (2018) 17:162–73. 10.1016/S1474-4422(17)30470-2 29275977

[11] Wingerchuk DM Banwell B Bennett JL Cabre P Carroll W Chitnis T. International consensus diagnostic criteria for neuromyelitis optica spectrum disorders. Neurology. (2015) 85:177–89. 10.1212/WNL.0000000000001729 26092914 PMC4515040

[12] Saraux A Pers JO Devauchelle-Pensec V. Treatment of primary Sjogren syndrome. Nat Rev Rheumatol. (2016) 12:456–71. 10.1038/nrrheum.2016.100 27411907

[13] Ramos-Casals M Brito-Zeron P Bombardieri S Bootsma H De Vita S Dorner T. EULAR recommendations for the management of Sjogren's syndrome with topical and systemic therapies. Ann Rheum Dis. (2019). 10.1136/annrheumdis-2019-216114 31672775

[14] Gottenberg JE Cinquetti G Larroche C Combe B Hachulla E Meyer O. Efficacy of rituximab in systemic manifestations of primary Sjogren's syndrome: results in 78 patients of the AutoImmune and Rituximab registry. Ann Rheum Dis. (2013) 72:1026–31. 10.1136/annrheumdis-2012-202293 23264337

[15] Verstappen GM van Nimwegen JF Vissink A Kroese FGM Bootsma H. The value of rituximab treatment in primary Sjogren's syndrome. Clin Immunol. (2017) 182:62–71. 10.1016/j.clim.2017.05.002 28478105

[16] Acar-Denizli N Kostov B Ramos-Casals M Consortium SBD. The Big Data Sjögren Consortium: a project for a new data science era. Clin Exp Rheumatol. (2019) 37:19–23. 31464669

[17] Seror R Theander E Brun JG Ramos-Casals M Valim V Dorner T. Validation of EULAR primary Sjogren's syndrome disease activity (ESSDAI) and patient indexes (ESSPRI). Ann Rheum Dis. (2015) 74:859–66. 10.1136/annrheumdis-2013-204615 24442883

[18] Held F Kalluri SR Berthele A Klein AK Reindl M Hemmer B. Frequency of myelin oligodendrocyte glycoprotein antibodies in a large cohort of neurological patients. Mult Scler J Exp Transl Clin. (2021) 7:20552173211022767. 10.1177/20552173211022767 34262784 PMC8246507

[19] Goh C Phal PM Desmond PM. Neuroimaging in acute transverse myelitis. Neuroimaging Clin N Am. (2011) 21:951–73. 10.1016/j.nic.2011.07.010 22032509

[20] Trebst C Raab P Voss EV Rommer P Abu-Mugheisib M Zettl UK. Longitudinal extensive transverse myelitis–it's not all neuromyelitis optica. Nat Rev Neurol. (2011) 7:688–98. 10.1038/nrneurol.2011.176 22045269

[21] Escudero D Latorre P Codina M Coll-Canti J Coll J editors. Central Nervous System Disease in Sjögren's Syndrome. Annales de Médecine Interne. Paris: Masson (1995). 7653943

[22] Gono T Kawaguchi Y Katsumata Y Takagi K Tochimoto A Baba S. Clinical manifestations of neurological involvement in primary Sjogren's syndrome. Clin Rheumatol. (2011) 30:485–90. 10.1007/s10067-010-1458-7 20393864

[23] Jamilloux Y Magy L Hurtevent JF Gondran G de Seze J Launay D. Immunological profiles determine neurological involvement in Sjogren's syndrome. Eur J Intern Med. (2014) 25:177–81. 10.1016/j.ejim.2013.10.005 24176941

[24] Massara A Bonazza S Castellino G Caniatti L Trotta F Borrelli M. Central nervous system involvement in Sjogren's syndrome: unusual, but not unremarkable–clinical, serological characteristics and outcomes in a large cohort of Italian patients. Rheumatology. (2010) 49:1540–9. 10.1093/rheumatology/keq111 20444860

[25] Moreira I Teixeira F Martins Silva A Vasconcelos C Farinha F Santos E. Frequent involvement of central nervous system in primary Sjogren syndrome. Rheumatol Int. (2015) 35:289–94. 10.1007/s00296-014-3097-9 25056402

[26] Ye W Chen S Huang X Qin W Zhang T Zhu X. Clinical features and risk factors of neurological involvement in Sjogren's syndrome. BMC Neurosci. (2018) 19:26. 10.1186/s12868-018-0427-y 29703151 PMC5924492

[27] Mauch E Völk C Kratzsch G Krapf H Kornhuber HH Laufen H. Neurological and neuropsychiatric dysfunction in primary Sjögren's syndrome. Acta Neurol Scand. (2009) 89:31–5. 10.1111/j.1600-0404.1994.tb01629.x 8178625

[28] Solomon DH Kavanaugh AJ Schur PH. Evidence-based guidelines for the use of immunologic tests: antinuclear antibody testing. Arthritis Rheum. (2002) 47:434–44. 10.1002/art.10561 12209492

[29] Szmyrka-Kaczmarek M Pokryszko-Dragan A Pawlik B Gruszka E Korman L Podemski R. Antinuclear and antiphospholipid antibodies in patients with multiple sclerosis. Lupus. (2011) 21:412–20. 10.1177/0961203311427550 22074845

[30] Tang WQ Wei SH. Primary Sjogren's syndrome related optic neuritis. Int J Ophthalmol. (2013) 6:888–91. 24392343 10.3980/j.issn.2222-3959.2013.06.26 PMC3874534

[31] Butryn M Neumann J Rolfes L Bartels C Wattjes MP Mahmoudi N. Clinical, radiological, and laboratory features of spinal cord involvement in primary Sjogren's syndrome. J Clin Med. (2020) 9:1482. 10.3390/jcm9051482 32423153 PMC7290729

[32] Hong X Wang X Rang X Yin X Zhang X Wang R. The shared mechanism and candidate drugs of multiple sclerosis and Sjogren's syndrome analyzed by bioinformatics based on GWAS and transcriptome data. Front Immunol. (2022) 13:857014. 10.3389/fimmu.2022.857014 35356004 PMC8959321

[33] Marrie RA Horwitz RI. Emerging effects of comorbidities on multiple sclerosis. Lancet Neurol. (2010) 9:820–8. 10.1016/S1474-4422(10)70135-6 20650403

[34] Marrie RA Reider N Cohen J Stuve O Sorensen PS Cutter G. A systematic review of the incidence and prevalence of autoimmune disease in multiple sclerosis. Mult Scler. (2015) 21:282–93. 10.1177/1352458514564490 25533299 PMC4429166

[35] Magyari M Sorensen PS. Comorbidity in multiple sclerosis. Front Neurol. (2020) 11:851. 10.3389/fneur.2020.00851 32973654 PMC7473304

[36] Fisher BA Brown RM Bowman SJ Barone F. A review of salivary gland histopathology in primary Sjogren's syndrome with a focus on its potential as a clinical trials biomarker. Ann Rheum Dis. (2015) 74:1645–50. 10.1136/annrheumdis-2015-207499 26034044

[37] Gorson KC Ropper AH. Positive salivary gland biopsy, Sjogren syndrome, and neuropathy: clinical implications. Muscle Nerve. (2003) 28:553–60. 10.1002/mus.10470 14571456

[38] Morreale M Marchione P Giacomini P Pontecorvo S Marianetti M Vento C. Neurological involvement in primary sjögren syndrome: a focus on central nervous system. PLoS ONE. (2014) 9:e0084605. 10.1371/journal.pone.0084605 24465419 PMC3896357

[39] Manzo C Martinez-Suarez E Kechida M Isetta M Serra-Mestres J. Cognitive function in primary sjogren's syndrome: a systematic review. Brain Sci. (2019) 9:85. 10.3390/brainsci9040085 30991679 PMC6523842

[40] Riega-Torres JCL Trevino-Castro MA Hernandez-Galarza IJ Garza-Martinez MJ Vera-Pineda R Cardenas-de la Garza JA. Cognitive dysfunction in Sjogren's syndrome using the montreal cognitive assessment questionnaire and the automated neuropsychological assessment metrics: a cross-sectional study. Int J Rheum Dis. (2020) 23:1019–23. 10.1111/1756-185X.13889 32608054

